# Morphological and phylogenetic evidence for recognition of two new species of *Hyphoderma* (Basidiomycota) from southern China, with a key to all Chinese *Hyphoderma*

**DOI:** 10.3897/mycokeys.83.69909

**Published:** 2021-09-20

**Authors:** Qian-Xin Guan, Yi-Fei Li, Chang-Lin Zhao

**Affiliations:** 1 Key Laboratory for Forest Resources Conservation and Utilization in the Southwest Mountains of China, Ministry of Education, Southwest Forestry University, Kunming 650224, China Southwest Forestry University Kunming China; 2 Yunnan Academy of Biodiversity, Southwest Forestry University, Kunming 650224, China Southwest Forestry University Kunming China; 3 College of Biodiversity Conservation, Southwest Forestry University, Kunming 650224, China Southwest Forestry University Kunming China

**Keywords:** Corticioid fungi, diversity, Hyphodermataceae, molecular phylogeny, taxonomy, Yunnan Province

## Abstract

Wood-inhabiting fungi play crucial roles as decomposers in forest ecosystems and, in this study, two new wood-inhabiting corticioid fungi, *Hyphodermapuerense* and *H.tenuissimum***spp. nov.**, are proposed, based on a combination of morphological features and molecular evidence. *Hyphodermapuerense* is characterised by effused basidiomata with smooth to floccose hymenial surface, a monomitic hyphal system with clamped generative hyphae and ellipsoid basidiospores. *Hyphodermatenuissimum* is characterised by resupinate basidiomata with tuberculate to minutely-grandinioid hymenial surface, septate cystidia and cylindrical to allantoid basidiospores. Sequences of ITS and nLSU rRNA markers of the studied samples were generated and phylogenetic analyses were performed with Maximum Likelihood, maximum parsimony and Bayesian Inference methods. These analyses showed that the two new species clustered into *Hyphoderma*, in which *H.puerense* grouped with *H.moniliforme* and *H.tenuissimum* formed a singleton lineage. In addition, an identification key to Chinese *Hyphoderma* is provided.

## Introduction

Fungi are eukaryotic microorganisms that play fundamental ecological roles as decomposers and mutualists of plants and animals. They drive carbon cycling in forest soils, mediate mineral nutrition of plants and alleviate carbon limitations of other soil organisms (Tedersoo et al. 2014). Fungi form an ecologically important branch of the tree of life, based on their distinct and diverse characters (James et al. 2020).

*Hyphoderma* Wallr. was typified by *H.setigerum* (Fr.) Donk (Donk 1957) and the genus is characterised by resupinate to effuse-reflexed basidiomata of ceraceous consistency and a smooth to tuberculate or hydnoid hymenophore. *Hyphoderma* species are characterised by a monomitic (rarely dimitic) hyphal structure with clamp connections on generative hyphae, presence of cystidia or not, suburniform to subcylindrical to cylindrical basidia and ellipsoid to subglobose, smooth, thin-walled basidiospores (Wallroth 1833; Bernicchia and Gorjón 2010). Currently, about 105 species have been accepted in *Hyphoderma* worldwide (Donk 1957; Nakasone 2008; Wu et al. 2010; Baltazar et al. 2016; Martín et al. 2018; Guan and Zhao 2021a, 2021b; Ma et al. 2021). Index Fungorum (http://www.indexfungorum.org; accessed on 16 July 2021) and MycoBank (https://www.mycobank.org; accessed on 16 July 2021) register 199 specific and infraspecific names in *Hyphoderma*.

*Hyphoderma* has been studied using molecular data, particularly the internal transcribed spacer (ITS) region and the large subunit nuclear ribosomal RNA gene (nLSU). Larsson (2007) showed that *H.obtusum* J. Erikss. and *H.setigerum* clustered into the Meruliaceae Rea and formed a sister taxon to *Hypochniciumpolonense* (Bres.) Å. Strid. Telleria et al. (2012) proposed a new species, *Hyphodermamacaronesicum* Tellería, M. Dueñas, Beltrán-Tej., Rodr.-Armas & M.P. Martín and then discussed the relationships with the closely-related taxa in *Hyphoderma*. Research into the *Hyphodermasetigerum* complex showed that *H.pinicola* Yurch. & Sheng H. Wu represented a fifth species in this complex (Yurchenko and Wu 2014b). A revised family-level classification of the Polyporales revealed that four *Hyphoderma* species grouped into the residual polyporoid clade, belonging to Hyphodermataceae in that they grouped with three related genera in Meripilaceae: *Meripilus* P. Karst., *Physisporinus* P. Karst. and *Rigidoporus* Murrill (Justo et al. 2017).

In this study, two undescribed species of corticioid fungi from forest ecosystems were collected in the Yunnan Province, China. We present morphological and molecular phylogenetic evidence that support the recognition of two new species in *Hyphoderma*, based on the nuclear ribosomal internal transcribed spacer region (ITS1, 5.8S and ITS2) and the nuclear ribosomal nLSU (28S) gene.

## Materials and methods

### Morphology

The studied specimens are deposited at the Herbarium of Southwest Forestry University (SWFC), Kunming, Yunnan Province, P.R. China. Macromorphological descriptions are based on field notes and photos captured in the field and lab. Colour terminology follows Petersen (Petersen 1996). Micromorphological data were obtained from the dried specimens when observed under a light microscope following Dai (2012). The following abbreviations are used: **KOH** = 5% potassium hydroxide water solution, **CB** = Cotton Blue, **CB–** = acyanophilous, **IKI** = Melzer’s Reagent, **IKI–** = both inamyloid and indextrinoid, **L** = mean spore length (arithmetic average for all spores), **W** = mean spore width (arithmetic average for all spores), **Q** = variation in the L/W ratios between the specimens studied and **n** = a/b (number of spores (a) measured from given number (b) of specimens).

### Molecular phylogeny

The CTAB rapid plant genome extraction kit-DN14 (Aidlab Biotechnologies Co., Ltd, Beijing) was used to obtain genomic DNA from the dried specimens following the manufacturer’s instructions (as done in Zhao and Wu 2017). The nuclear ribosomal ITS region was amplified with the primers ITS5 and ITS4 (White et al. 1990). The nuclear ribosomal LSU gene was amplified with the primers LR0R and LR7 (Vilgalys and Hester 1990; Rehner and Samuels 1994). The PCR procedure for ITS was as follows: initial denaturation at 95 °C for 3 min followed by 35 cycles at 94 °C for 40 s, 58 °C for 45 s and 72 °C for 1 min and a final extension of 72 °C for 10 min. The PCR procedure for nLSU was as follows: initial denaturation at 94 °C for 1 min followed by 35 cycles at 94 °C for 30 s, 48 °C for 1 min and 72 °C for 1.5 min and a final extension of 72 °C for 10 min. The PCR products were purified and sequenced at Kunming Tsingke Biological Technology Limited Company, Kunming, Yunnan Province, P.R. China. All newly-generated sequences were deposited in NCBI GenBank (Table 1).

**Table 1.** List of species, specimens and GenBank accession numbers of sequences used in this study.

Species name	Specimen No.	GenBank accession No.		References
		ITS	LSU	
* Climacocystis borealis *	FD-31	KP135308	KP135210	Justo et al. (2017)
* Diplomitoporus crustulinus *	FD-137	KP135299	KP135211	Justo et al. (2017)
* Hyphoderma amoenum *	USO 286622	HE577030	—	Telleria et al. (2012)
* H. assimile *	CBS 125852	MH863808	MH875272	Vu et al. (2019)
* H. cremeoalbum *	NH 11538	DQ677492	DQ677492	Larsson (2007)
* H. crystallinum *	CLZhao 9338	MW917161	MW913414	Guan and Zhao (2021a)
	CLZhao 9374	MW917162	MW913415	Guan and Zhao (2021a)
	CLZhao 10224	MW917163	MW913416	Guan and Zhao (2021a)
	CLZhao 11723	MW917164	MW913417	Guan and Zhao (2021a)
	CLZhao 15841	MW917165	MW913418	Guan and Zhao (2021a)
	CLZhao 18459	MW917166	MW913419	Guan and Zhao (2021a)
* H. definitum *	GEL 2898	—	AJ406509	Yurchenko and Wu (2014)
	NH 12266	DQ677493	DQ677493	Larsson (2007)
* H. fissuratum *	CLZhao 6731	MT791331	MT791335	Ma et al. (2021)
	CLZhao 6726	MT791330	MT791334	Ma et al. (2021)
* H. floccosum *	CLZhao 17129	MW301683	MW293733	Guan and Zhao (2021b)
	CLZhao 17296	MW301686	MW293736	Guan and Zhao (2021b)
	CLZhao 16492	MW301688	MW293734	Guan and Zhao (2021b)
	CLZhao 17215	MW301687	MW293735	Guan and Zhao (2021b)
* H. granuliferum *	KHL 12561	JN710545	JN710545	Yurchenko and Wu (2014)
* H. incrustatum *	KHL 6685	—	AY586668	Yurchenko and Wu (2014)
* H. litschaueri *	NH 7603	DQ677496	DQ677496	Larsson (2007)
	FP-101740-Sp	KP135295	KP135219	Floudas and Hibbett (2015)
* H. macaronesicum *	MA:Fungi:16099	HE577027	—	Yurchenko and Wu (2014)
	TFC:Mic.15981	HE577028	—	Yurchenko and Wu (2014)
* H. medioburiense *	NH 10950	DQ677497	DQ677497	Larsson (2007)
* H. membranaceum *	CLZhao 5844	MW917167	MW913420	Guan and Zhao (2021a)
	CLZhao 6971	MW917168	MW913421	Guan and Zhao (2021a)
* H. microporoides *	CLZhao 6857	MW917169	MW913422	Guan and Zhao (2021a)
	CLZhao 8695	MW917170	MW913422	Guan and Zhao (2021a)
* H. moniliforme *	Wu 0211–42	KC928282	—	Yurchenko and Wu (2015)
	Wu 0211–46	KC928284	KC928285	Yurchenko and Wu (2015)
* H. mopanshanense *	CLZhao 6498	MT791329	MT791333	Ma et al. (2021)
	CLZhao 6493	MT791328	MT791332	Ma et al. (2021)
* H. nemorale *	TNM F3931	KJ885183	KJ885184	Yurchenko and Wu (2015)
	Wu 9508–14	KC928280	KC928281	Yurchenko and Wu (2015)
* H. nudicephalum *	Wu 9307–29	AJ534269	—	Nilsson et al. (2003)
	Wu 9508–225	AJ534268	—	Nilsson et al. (2003)
* H. obtusiforme *	KHL 1464	JN572909	—	Yurchenko and Wu (2014)
	KHL 11105	JN572910	—	Yurchenko and Wu (2014)
* H. obtusum *	JS 17804	—	AY586670	Yurchenko and Wu (2014)
* H. occidentale *	KHL 8469	—	AY586674	Yurchenko and Wu (2014)
	KHL 8477	DQ677499	DQ677499	Larsson (2007)
* H. paramacaronesicum *	MA:Fungi:87736	KC984399	—	Martín et al. (2018)
	MA:Fungi:87737	KC984405	—	Martín et al. (2018)
* H. pinicola *	Wu 0108–32	KJ885181	KJ885182	Yurchenko and Wu (2014)
	Wu 0108–36	KC928278	KC928279	Yurchenko and Wu (2014)
* H. prosopidis *	E09/58–9	HE577029	—	Yurchenko and Wu (2015)
*** H. puerense ***	CLZhao 9476*	**MW443045**	—	**Present study**
	**CLZhao 9583**	**MW443046**	**MW443051**	**Present study**
* H. roseocremeum *	NH 10545	—	AY586672	Yurchenko and Wu (2014)
* H. setigerum *	FCUG 1200	AJ534273	—	Nilsson et al. (2003)
* H. setigerum *	FCUG 1688	AJ534272	—	Nilsson et al. (2003)
* H. sinense *	CLZhao 7963	MW301679	MW293730	Guan and Zhao (2021b)
	CLZhao 17811	MW301682	MW293732	Guan and Zhao (2021b)
	CLZhao 7981	MW301680	MW293731	Guan and Zhao (2021b)
*Hyphoderma* sp.	KUC20121102–21	KJ668522	—	Unpublished
	KUC11052	KJ714002	—	Jang et al. (2015)
	Wu 0311–25	KR868735	—	Unpublished
	Wu 0310–6	KR868736	—	Unpublished
	Wu 0808–87	KR868737	—	Unpublished
	GEL3689	DQ340327	—	Unpublished
* H. subsetigerum *	Wu 9304–18	AJ534277	—	Nilsson et al. (2003)
	Wu 9202–15	AJ534278	—	Nilsson et al. (2003)
* H. subsetigerum *	HHB11620	GQ409521	—	Yurchenko and Wu (2014)
	CFMR MJL1536	GQ409522	—	Yurchenko and Wu (2014)
*** H. tenuissimum ***	**CLZhao 6930**	**MW443047**	**MW443052**	**Present study**
	**CLZhao 7003**	**MW443048**	**MW443053**	**Present study**
	CLZhao 7221*	**MW443049**	**MW443054**	**Present study**
	**CLZhao 16210**	**MW443050**	**MW443055**	**Present study**
* H. transiens *	NH 12304	DQ677504	DQ677504	Larsson (2007)
* H. variolosum *	CBS 734.91	MH862320	MH873992	Vu et al. (2019)
	CBS 735.91	MH862321	MH873993	Vu et al. (2019)
* Hypochnicium erikssonii *	NH 9635	—	DQ677508	Larsson (2007)
* H. geogenium *	NH 10910	—	DQ677509	Larsson (2007)
	MA-Fungi 48308	FN552534	JN939576	Telleria et al. (2010)
* H. michelii *	MA-Fungi 79155	NR119742	NG060635	Telleria et al. (2010)
* H. punctulatum *	FP101698sp	KY948827	KY948860	Justo et al. (2017)
* H. sphaerosporum *	RLG15138sp	KY948803	KY948861	Justo et al. (2017)
* H. wakefieldiae *	MA-Fungi 7675	FN552531	JN939577	Telleria et al. (2010)
* Physisporinus subcrocatus *	Dai 15917	KY131870	KY131926	Wu et al. (2017)
* P. subcrocatus *	Dai 12800	KY131869	KY131925	Wu et al. (2017)
* P. tibeticus *	Cui 9588	KY131873	KY131929	Wu et al. (2017)
	Cui 9518	KY131872	KY131928	Wu et al. (2017)
* Rigidoporus eminens *	Dai 17200	MT279690	MT279911	Wu et al. (2017)
* R. undatus *	Miettinen-13591	KY948731	KY948870	Justo et al. (2017)

New species is shown in bold; * type material.

**Figure 1.**
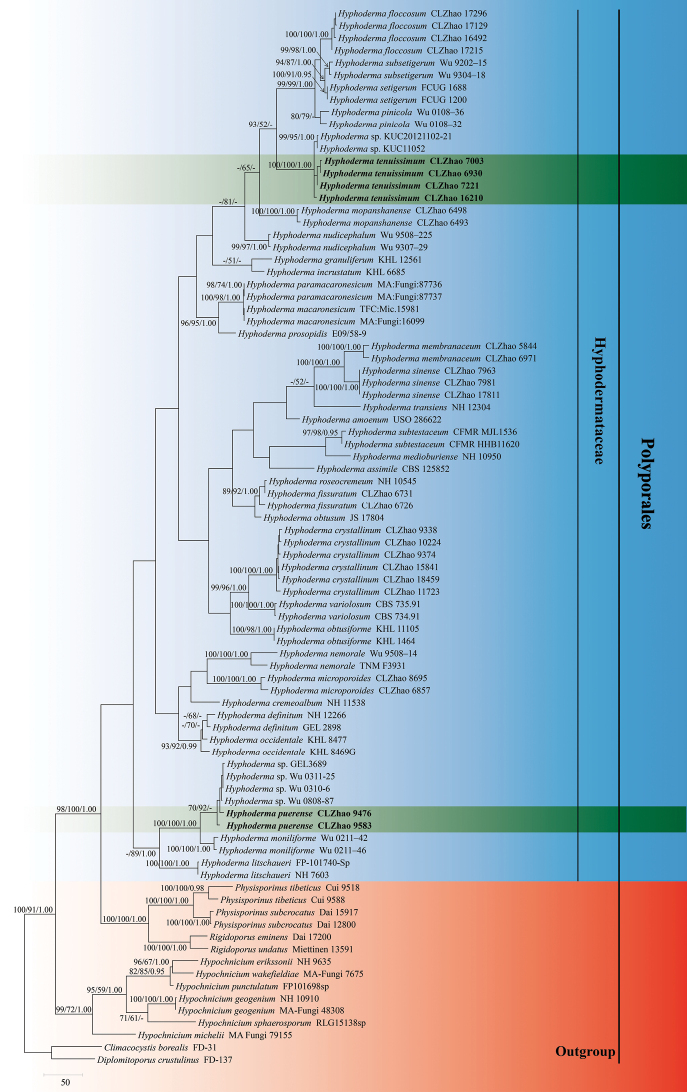
Maximum parsimony strict consensus tree illustrating the phylogeny of the two new species and related species in *Hyphoderma*, based on ITS1+5.8S+ITS2+nLSU sequences. Branches are labelled with maximum likelihood bootstrap values > 70%, parsimony bootstrap values > 50% and Bayesian posterior probabilities > 0.95, respectively.

The sequences were aligned in MAFFT version 7 (Katoh et al. 2019) using the G-INS-i strategy. The alignment was adjusted manually using AliView version 1.27 (Larsson 2014). Each dataset was aligned separately at first and then the ITS1, 5.8S, ITS2 and nLSU regions were combined with Mesquite version 3.51. The combined dataset was deposited in TreeBASE (submission ID 28564). *Climacocystisborealis* (Fr.) Kotl. and Pouzar and *Diplomitoporuscrustulinus* (Bres.) Domański were selected as outgroup (Fig. 1) as inspired by a previous study (Justo et al. 2017).

Maximum parsimony analysis in PAUP* version 4.0a169 (http://phylosolutions.com/paup-test/) was applied to the combined ITS1+5.8S+ITS2+nLSU dataset. All characters were equally weighted and gaps were treated as missing data. Trees were inferred using the heuristic search option with TBR branch swapping and 1,000 random sequence additions. Max-trees were set to 5,000, branches of zero length were collapsed and all parsimonious trees were saved. Clade robustness was assessed using bootstrap (BT) analysis with 1,000 pseudoreplicates (Felsenstein 1985). Descriptive tree statistics – tree length (TL), composite consistency index (CI), composite retention index (RI), composite rescaled consistency index (RC) and composite homoplasy index (HI) – were calculated for each maximum parsimonious tree generated. The combined dataset was also analysed using Maximum Likelihood (ML) in RAxML-HPC2 through the CIPRES Science Gateway (Miller et al. 2012). Branch support (BS) for the ML analysis was determined by 1,000 bootstrap pseudoreplicates.

MrModeltest 2.3 (Nylander 2004) was used to determine the best-fit evolution model for each dataset (ITS1+5.8S+ITS2+nLSU) for Bayesian Inference (BI). BI was calculated with MrBayes version 3.2.7a (Ronquist et al. 2012). Four Markov chains were run for two runs from random starting trees for 3 million generations (Fig. 1). The first 25% of all generations was discarded as burn-in. A majority rule consensus tree was computed from the remaining trees. Branches were considered as significantly supported if they received a maximum likelihood bootstrap support value (BS) of > 70%, a maximum parsimony bootstrap support value (BT) of > 70% or a Bayesian posterior probability (BPP) of > 0.95.

## Results

### Molecular phylogeny

The ITS1+5.8S+ITS2+nLSU dataset comprised sequences from 86 fungal specimens representing 46 taxa. The dataset had an aligned length of 2,034 characters, of which 1,360 characters were constant, 131 were variable and parsimony-uninformative and 543 (35%) were parsimony-informative. Maximum parsimony analysis yielded 108 equally parsimonious trees (TL = 3,317, CI = 0.3361, HI = 0.6946, RI = 0.7051 and RC = 0.2370). The best model of nucleotide evolution for the ITS1+5.8S+ITS2+nLSU dataset estimated and applied in the Bayesian analysis was found to be GTR+I+G. Bayesian analysis and ML analysis resulted in a similar topology as in the MP analysis. The Bayesian analysis had an average standard deviation of split frequencies = 0.008952 (BI) and the effective sample size (ESS) across the two runs is double the average ESS (avg. ESS) = 1,771. The Bayesian tree is shown here (Fig. 1).

The phylogram inferred from ITS1+5.8S+ITS2+nLSU sequences (Fig. 1) highlights the two undescribed species in *Hyphoderma*; *H.puerense* as a sister to *H.moniliforme* and *H.tenuissimum* that forms an independent monophyletic lineage (100% parsimony bootstrap support, 100% likelihood bootstrap support and 1.00 BPP).

### Taxonomy

#### Hyphoderma puerense

Taxon classificationFungiPolyporalesMeruliaceae

C.L. Zhao & Q.X. Guan sp. nov.

73F48FA2-9D28-59A2-A6AC-AE1A438B6452

838411

Figs 2 , 3

##### Holotype.

China. Yunnan Province, Puer, Jingdong County, Huilianghe Village, GPS co-ordinates 24°04'45"N, 100°56'32"E, altitude 1246 m a.s.l., on fallen angiosperm branch, leg. C.L. Zhao, 4 January 2019, CLZhao 9476 (SWFC).

##### Etymology.

*puerense* (Lat.): referring to the locality (Puer) of the specimens.

##### Description.

Basidioma annual, resupinate, adnate, byssoid, without odour and taste when fresh, up to 15 cm long, 3 cm wide, 100–260 µm thick. Hymenial surface smooth to floccose, cream when fresh, cream to slightly buff on drying. Margin sterile, thinning out, narrow, cream.

Hyphal system monomitic, generative hyphae with clamps, colourless, thick-walled, frequently branched, interwoven, 2.5–4.5 µm in diameter; IKI-, CB-; tissues unchanged in KOH; subhymenial hyphae densely covered by crystals.

**Figure 2.**
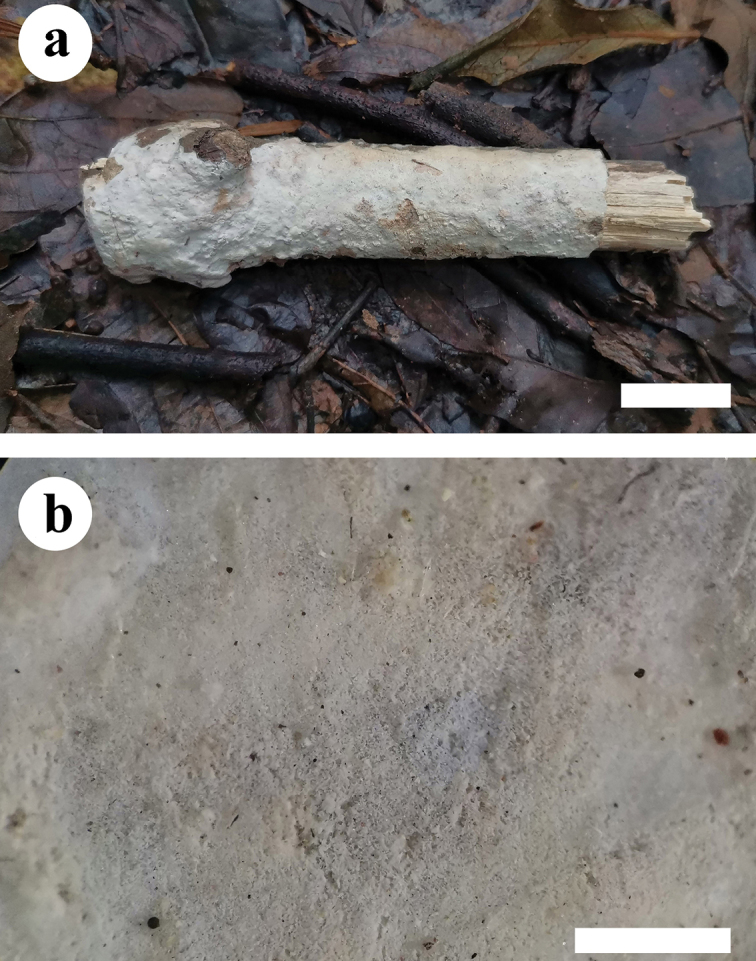
Basidiomata of *Hyphodermapuerense* (holotype). Scale bars: 2 cm (**a**); 1 mm (**b**).

Cystidia tubular, encrusted with small crystals, 25–97 × 5.5–9.5 µm.

Basidia clavate to subcylindrical, slightly constricted in the middle to somewhat sinuous, with 4 sterigmata and a basal clamp, 20–30 × 4.5–6 µm.

**Figure 3.**
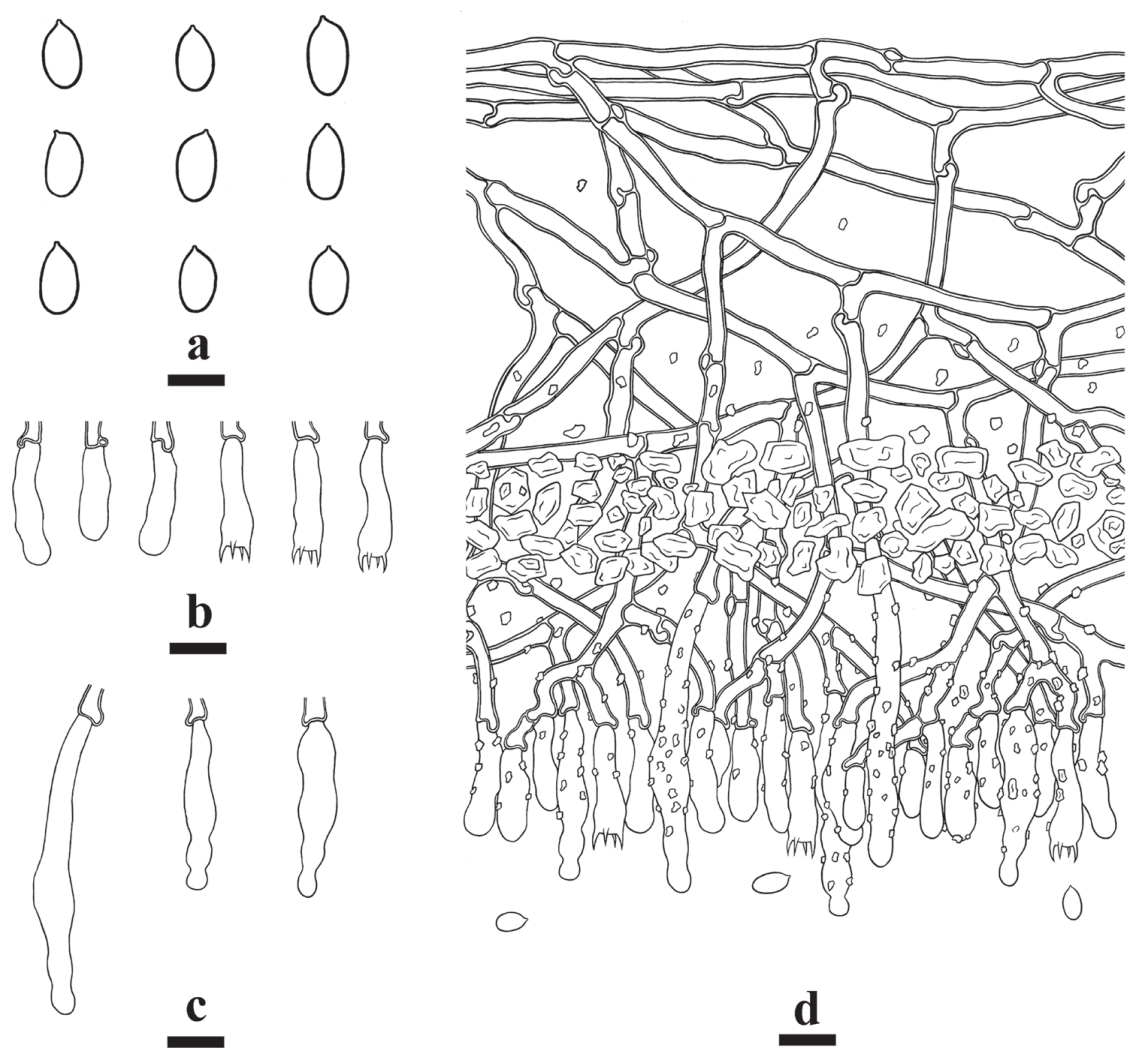
Microscopic structures of *Hyphodermapuerense* (holotype) **a** basidiospores **b** basidia and basidioles **c** cystidia **d** a section of hymenium. Scale bars: 5 µm (**a**); 10 µm (**b–d**).

Basidiospores ellipsoid, colourless, thin-walled, smooth, IKI-, CB-, (5.5–)6–7.5(–8) × 3–4.5(–5) µm, L = 6.53 µm, W = 3.71 µm, Q = 1.73–1.79 (n = 60/2).

##### Habitat and ecology.

Climate of the sample collection site is subtropical monsoon climate area, the forest type is evergreen angiosperm forest and samples were collected on fallen angiosperm branches.

**Figure 5.**
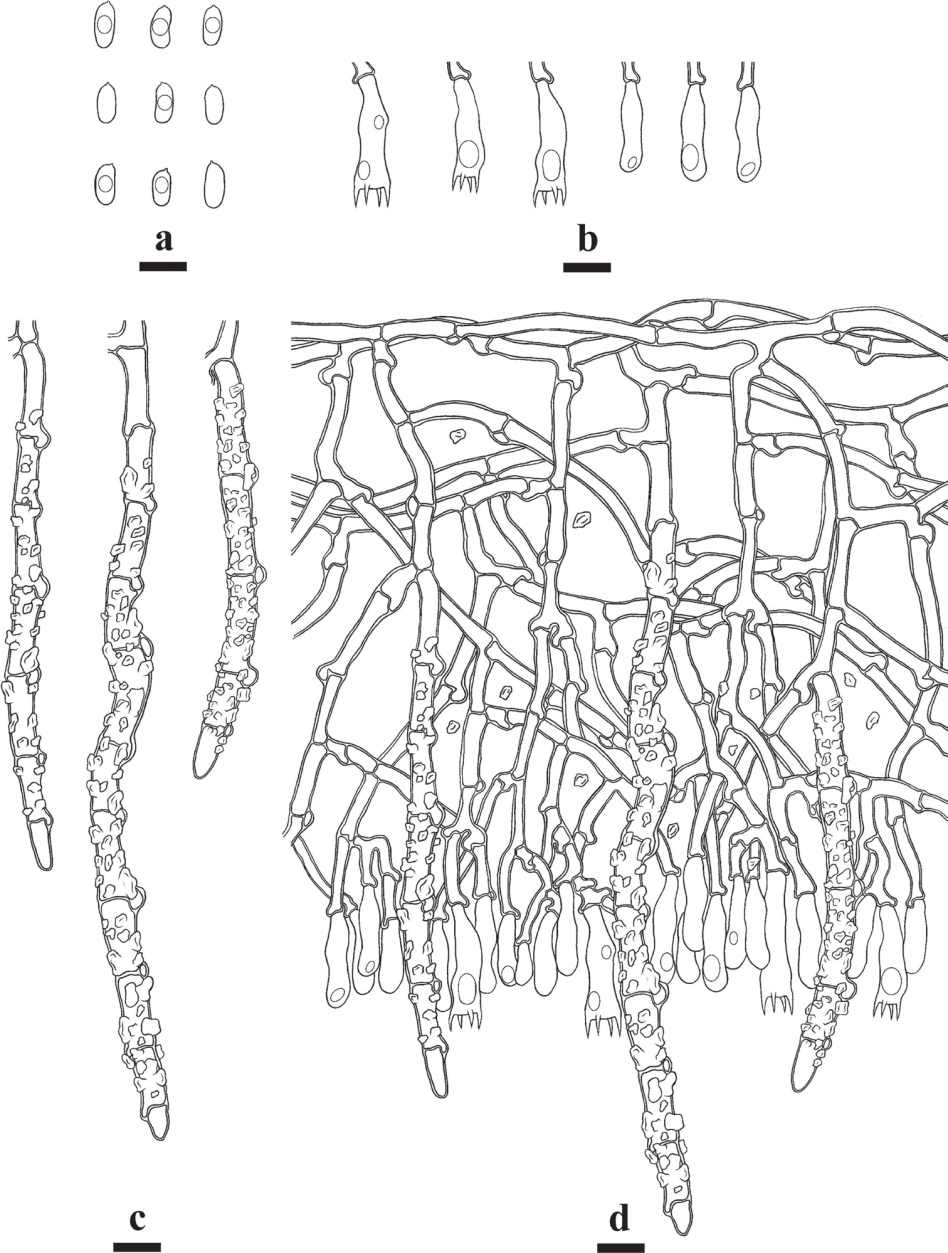
Microscopic structures of *Hyphodermatenuissimum* (holotype) **a** basidiospores **b** basidia and basidioles **c** cystidia **d** a section of hymenium. Scale bars: 10 µm (**a–d**).

##### Additional specimens examined.

China. Yunnan Province, Puer, Jingdong County, Huilianghe Village, GPS co-ordinates 24°04'45"N, 100°56'32"E, altitude 1246 m a.s.l., on fallen angiosperm branch, leg. C.L. Zhao, 5 January 2019, CLZhao 9583 (SWFC).

#### Hyphoderma tenuissimum

Taxon classificationFungiPolyporalesMeruliaceae

C.L. Zhao & Q.X. Guan sp. nov.

523885D7-51A5-5383-B36E-DB68F4E670D0

838412

Figs 4 , 5

##### Holotype.

China. Yunnan Province, Chuxiong, Zixishan Forestry Park, GPS co-ordinates 25°01'26"N, 101°24'37"E, altitude 2313 m a.s.l., on fallen angiosperm branch, leg. C.L. Zhao, 1 July 2018, CLZhao 7221 (SWFC).

##### Etymology.

*tenuissimum* (Lat.): referring to the thin basidiomata.

##### Description.

Basidioma annual, resupinate, adnate, membranaceous when fresh, hard membranaceous upon drying, up to 20 cm long, 3 cm wide, 30–100 µm thick. Hymenial surface tuberculate to minutely-grandinioid, slightly buff when fresh, buff upon drying, cracking. Margin sterile, slightly buff, 1 mm wide.

**Figure 4.**
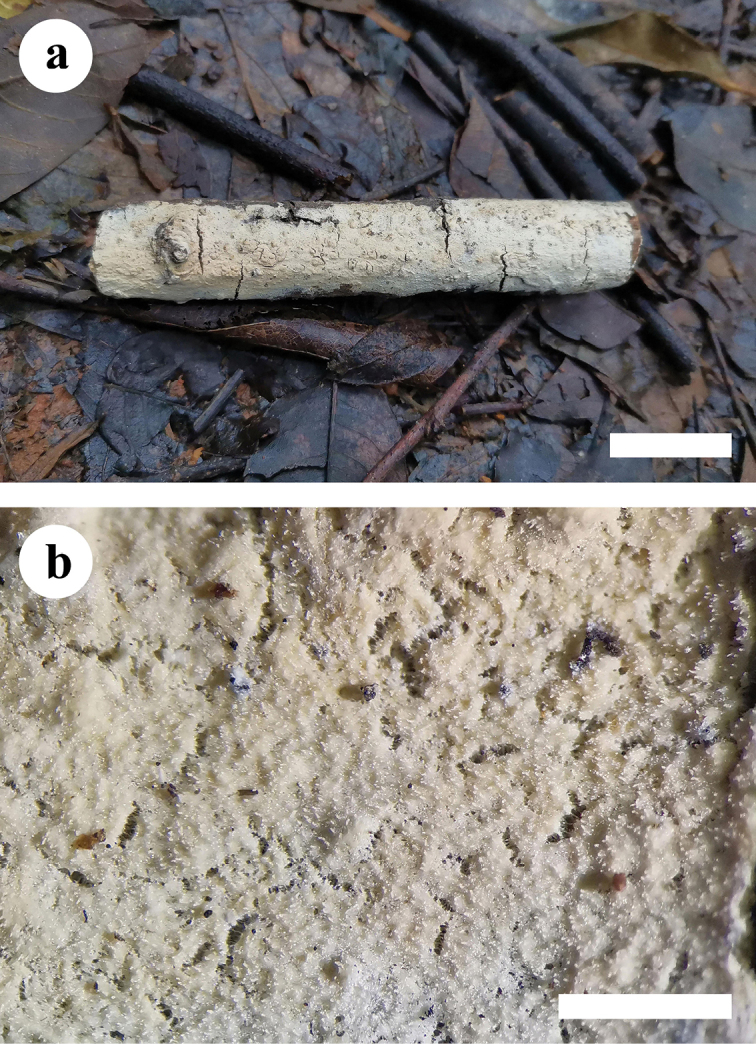
Basidiomata of *Hyphodermatenuissimum* (holotype). Scale bars: 2 cm (**a**); 1 mm (**b**).

Hyphal system monomitic, generative hyphae with clamps, colourless, thick-walled, frequently branched, interwoven, 3–5 µm in diameter, IKI-, CB-; tissues unchanged in KOH.

Cystidia large, cylindrical, with 4–12 clamped septa, with abundant encrustations, 50–220 × 6.5–13 µm.

Basidia clavate to subcylindrical, constricted, somewhat sinuous, with 4 sterigmata and a basal clamp connection, 17–31 × 4.5–8 µm.

Basidiospores cylindrical, colourless, thin-walled, smooth, with oil drops inside, IKI–, CB–, 7–10.5(–11) × 3–4.5(–5) µm, L = 8.75 µm, W = 4.15 µm, Q = 2.02–2.18 (n = 120/4).

##### Habitat and ecology.

Climate of the sample collection site is subtropical monsoon climate area, the forest type is evergreen angiosperm forest and samples were collected on fallen angiosperm branches.

##### Additional specimens examined.

China. Yunnan Province, Chuxiong, Zixishan National Forestry Park, GPS co-ordinates 25°01'26"N, 101°24'37"E, altitude 2263 m a.s.l., on fallen angiosperm branch, leg. C.L. Zhao, 1 July 2018, CLZhao 6930, CLZhao 7003 (SWFC); Wenshan, Pingba Town, Wenshan National Nature Reserve, GPS co-ordinates 23°18'19"N, 104°42'47"E, altitude 1976 m a.s.l., on fallen angiosperm branch, leg. C.L. Zhao, 25 July 2019, CLZhao 16210 (SWFC).

## Discussion

In the present study, two new species, *Hyphodermapuerense* and *H.tenuissimum* are described, based on phylogenetic analyses and morphological characters.

Phylogenetically, the two new taxa were found to belong to *Hyphoderma*, in which *H.puerense* forms a sister species to *H.moniliforme* and *H.tenuissimum* forms an independent monophyletic lineage (100% BS, 100% BP and 1.00 BPP).

Morphologically, *Hyphodermapuerense* is similar to *H.obtusiforme* J. Erikss. & Å. Strid and *H.obtusum* in having a smooth hymenium, non-septate cylindrical cystidia and ellipsoid basidiospores. However, *H.obtusiforme* differs from *H.puerense* by both larger basidia (30–40 × 8–9 µm) and basidiospores (10–14 × 5–7 µm; Eriksson and Ryvarden 1975). *Hyphodermaobtusum* also differs from *H.puerense* by larger basidia (30–35 × 6–8 µm) and basidiospores (8–9 × 5–6.5 µm; Eriksson 1958). *Hyphodermapuerense* is similar to *H.roseocremeum* (Bres.) Donk in having smooth hymenium and non-septate cylindrical cystidia. However, *Hyphodermaroseocremeum* differs through the presence of larger basidiospores (8–12 × 3–4 µm; Bernicchia and Gorjón 2010).

Morphologically, *Hyphodermatenuissimum* is similar to *H.floccosum* C.L. Zhao & Q.X. Guan, *H.mopanshanense*, *H.nudicephalum* Gilb. & M. Blackw., *H.pinicola*, *H.setigerum* and *H.subsetigerum* Sheng H. Wu in having septocystidia and cylindrical basidiospores. However, *Hyphodermafloccosum* differs from *H.tenuissimum* by having a floccose hymenial surface and tubular cystidia (Guan and Zhao 2021b); *H.mopanshanense* is separated from *H.tenuissimum* by having porulose to pilose hymenial surface and smaller basidia (15–18.5 × 3–4.5 µm; Ma et al. 2021); *H.nudicephalum* differs from *H.tenuissimum* in the nature of the septocystidial apex (lacking encrustation; swollen up to 14 µm; Gilbertson and Blackwell 1988); *H.pinicola* is separated from *H.tenuissimum* by having basidia with two sterigmata and larger basidiospores (13–16 × 4–4.5 µm; Yurchenko and Wu 2014b); *H.setigerum* differs by having a combination of thin basidiomata with very long septocystidia (Bernicchia and Gorjón 2010); and *H.subsetigerum* differs from *H.tenuissimum* by having narrower basidia (20–30 × 4.5–5.5 µm) and smaller basidiospores (6–8 × 2.8–3.2 µm; Wu 1997).

Nilsson et al. (2003) highlighted the phylogeography of *Hyphodermasetigerum* (Basidiomycota) in the Northern Hemisphere in a study based on molecular analysis, morphological studies and crossing tests. Nine preliminary taxa were shown to exist inside the *H.setigerum* complex; in the present study, *H.tenuissimum* belongs to the *H.setigerum* complex, based on the morphological character of long septocystidia and phylogenetic evidence. A previous study indicated the importance of vicariance in the evolution of this species complex (Nilsson et al. 2003) and our study shows that the specimens of *H.tenuissimum* are collected in Zixishan National Forestry Park (GPS co-ordinates 25°01'26"N, 101°24'37"E), Chuxiong, Yunnan Province, China, which is distinct from *H.setigerum* s. str. (Norway: Oppland and Finland: Pohjois-Häme). The present samples of *H.subsetigerum* and *H.nudicephalum* were collected in Yunnan Province, China, but neither of these taxa groups together closely with *H.tenuissimum* (Fig. 1).

In the current phylogenetic tree, two partially annotated GenBank sequences (KJ668522 and KJ714002) of *Hyphoderma* sp. (South Korea) cluster closely with four sequences of the new species *Hyphodermatenuissimum*, although whether they really belong to this species remains to be assessed. It is certainly conceivable that they do, which would mean that *Hyphodermatenuissimum* has been collected and sequenced at least six times in Asia. Regarding the new taxon *H.puerense* (Fig. 1), four partially annotated GenBank sequences (KR868735, KR868736, KR868737 and DQ340327) form a reasonably well-supported clade together with our two specimens of *H.puerense.* We interpret this to mean that all six taxa represent *H.puerense*. All of the samples in this clade are from Asia, which supports the point of the importance of vicariance in the evolution in this genus.

### Key to 30 accepted species of *Hyphoderma* in China

**Table:** 

1	Cystidia absent	**2**
–	Cystidia present	**5**
2	Hymenial surface grandinioid	*** H. acystidiatum ***
–	Hymenial surface smooth	**3**
3	Basidiospores > 10.5 µm in length	*** H. densum ***
–	Basidiospores < 10.5 µm in length	**4**
4	Hymenophore cracked; basidiospores > 8.5 µm in length	*** H. fissuratum ***
–	Hymenophore uncracked; basidiospores < 8.5 µm in length	*** H. sibiricum ***
5	Hymenophore smooth	**6**
–	Hymenophore tuberculate, porulose, grandinioid or odontoid	**15**
6	Two types of cystidia present	**7**
–	One type of cystidia present	**8**
7	Moniliform cystidia absent	*** H. microcystidium ***
–	Moniliform cystidia present	*** H. sinense ***
8	Hymenophore uncracked	**9**
–	Hymenophore cracked	**11**
9	Basidiospores > 11 µm in length	*** H. definitum ***
–	Basidiospores < 11 µm in length	**10**
10	Basidiospores > 8.5 µm in length	*** H. microporoides ***
–	Basidiospores < 8.5 µm in length	*** H. puerense ***
11	Cystidia moniliform	**12**
–	Cystidia cylindrical	**13**
12	Basidiospores > 9 µm in length	*** H. litschaueri ***
–	Basidiospores < 9 µm in length	*** H. moniliforme ***
13	Basidiospores ellipsoid < 10 μm in length	*** H. rimulosum ***
–	Basidiospores cylindrical > 10 μm in length	**14**
14	Basidiospores > 12 µm in length	*** H. cremeum ***
–	Basidiospores < 12 µm in length	*** H. subclavatum ***
15	Hymenophore odontoid or grandinioid	**16**
–	Hymenophore tuberculate, porulose	**19**
16	Hymenophore odontoid	**17**
–	Hymenophore grandinioid	**18**
17	Basidiospores < 4.5 µm in width	*** H. transiens ***
–	Basidiospores > 4.5 µm in width	*** H. formosanum ***
18	Basidiospores larger 7–10.5 × 3–4.5 µm	*** H. tenuissimum ***
–	Basidiospores smaller 6–8 × 2.8–3.2 μm	*** H. subsetigerum ***
19	Cystidia of two types	**20**
–	Cystidia of one type	**23**
20	Septate cystidia absent	**21**
–	Septate cystidia present	**22**
21	Basidiospores < 4 µm in width	*** H. variolosum ***
–	Basidiospores > 4 µm in width	*** H. crystallinum ***
22	Basidia 2-sterigmata, basidiospores > 13 µm in length	*** H. pinicola ***
–	Basidia 4-sterigmata, basidiospores < 13 µm in length	*** H. floccosum ***
23	Septate cystidia present	**24**
–	Septate cystidia absent	**25**
24	Hymenophore porulose to pilose, basidia < 5 µm in width	*** H. mopanshanense ***
–	Hymenophore tuberculate, basidia > 5 µm in width	*** H. setigerum ***
25	Hymenophore porulose	*** H. obtusiforme ***
–	Hymenophore tuberculate, colliculose	***26***
26	Cystidia < 30 µm in length	*** H. cremeoalbum ***
–	Cystidia > 30 µm in length	**27**
27	Basidia > 30 µm in length	**28**
–	Basidia < 30 µm in length	**29**
28	Hymenophore cracking, cystidia < 10 µm in width	*** H. medioburiense ***
–	Hymenophore not cracking, cystidia > 10 µm in width	*** H. clavatum ***
29	Hymenophore colliculose	*** H. nemorale ***
–	Hymenophore tuberculate	*** H. membranaceum ***

## Supplementary Material

XML Treatment for Hyphoderma puerense

XML Treatment for Hyphoderma tenuissimum
